# School-based injury outcomes in children from a low-income setting: results from the pilot injury surveillance in Rawalpindi city, Pakistan

**DOI:** 10.1186/1756-0500-6-86

**Published:** 2013-03-07

**Authors:** Uzma Rahim Khan, Junaid A Bhatti, Nukhba Zia, Umar Farooq

**Affiliations:** 1 Department of Emergency Medicine, Aga Khan University, Stadium Road, Karachi, 74800, Pakistan; 2 Public Health Solutions Pakistan (Pvt.) Limited, Lahore, Pakistan; 3 Surgical Unit I, Department of Surgery, HolyFamilyHospital, Rawalpindi, Pakistan

**Keywords:** School injuries, Children, Pakistan

## Abstract

**Background:**

School-based injuries account for one in five unintentional childhood injuries. Little is known about the epidemiology of school-based injuries in low-income settings. The objective of our study was to compare emergency department (ED) outcomes of the school-based injuries with respect to age, sex, and injury mechanisms in a Pakistani urban setting.

**Findings:**

A pilot injury surveillance study was conducted at the EDs of three major tertiary-care hospitals of Rawalpindi city from July 2007 to June 2008 and included children of less than 15 years injured at school. The World Health Organization’s questionnaire for injury surveillance was used. There were 923 school injury cases. Mean age of children involved was 8.3 years (SD ± 3.3) with male female ratio 2.9:1. Most injuries occurred while playing 85.6% (n = 789); of which the most common mechanism was falls (n = 797, 86.4%). Nineteen of twenty cases were directly discharged home from the ED (N = 861). Compared to ED discharged cases, injury characteristics overrepresented in hospital admitted cases (n = 46) were age 10–14 years (65.2% vs. 40.9%, p = 0.005), male (88.6% vs. 25.9%), involved in educational activities (39.1% vs. 5.3%), injured from fire/heat (37.8% vs. 0.6%), had burns (39.5% vs. 0.9%) and head injuries (27.9% vs. 6.4%).

**Conclusion:**

Falls while playing are the commonest injury mechanism in school-based injuries reported in our ED sample. School officials need to prevent these injuries. Studying injury hazards present in school environment in Pakistan might facilitate developing specific prevention strategies.

## Findings

### Background

Childhood injuries are an important public health issue globally, affecting the low-and middle-income countries more than the high-income countries [1]. The combined work of The United Nations Children’s Fund (UNICEF) and the Alliance for Safe Children in five South and East Asian counties - Bangladesh, China, the Philippines, Thailand and Viet Nam – has shown that for every injury death of a child less than 18 years of age, 12 needed hospital admission or had permanent disability while 34 children required medical care or missed school/work [2]. Childhood injury can have devastating effects both on children and their families especially those from low socioeconomic group [1].

Injury causation is a multi-factorial phenomenon in which place of injury plays a very important role. Estimates have shown that home is the most common place where children are at the risk of injuries [1]. Other than homes, schools have also been found to be a common place of injuries [3-10] with a contribution of about 19% [11]. Children spend a significant amount of their time at schools [12] where many hazards such as defective equipment and improper playgrounds predispose them to different types of injuries. The contact of the students amongst themselves e.g. during sports-related activities and with the environment can act as possible contributing factors towards injury. The majority of injuries that happen in school are however non-fatal in nature [13].

In Pakistan, a low-income country with over 180 million inhabitants, every third person is aged less than 18 years. The two previous surveys on injuries from Pakistan were generalized and were not able to inform about characteristics of school-based injuries in children to guide preventive measures or research [14-16]. In order to develop effective injury prevention strategies for schools in Pakistan and similar low-income settings, it is important to understand the epidemiology of school injuries. The objective of the study is to characterize the school-based injuries that required Emergency department (ED) care with respect to age, sex, injury mechanisms and outcomes in a Pakistani urban setting. Notably, school-based injury characteristics were compared for two major outcomes i.e. discharged from ED and being admitted in hospital.

## Methods

### Study setting and population

The study setting was Rawalpindi city with an approximate population of 1.6 million in 2006–07. A pilot injury surveillance study was conducted at the Emergency Departments (EDs) of three major tertiary care hospitals of the city namely Holy Family Hospital, Benazir Bhutto Hospital (formerly called Rawalpindi General Hospital) and District Headquarter Hospital. These government-run facilities provided healthcare at nominal charges and serve mostly the low-income population; over two-third of population earns less than 2 US$ in Pakistan [17]. The total population of Pakistan below 15 years is around 43% [17]. The recent estimates indicated that about 84% of the population had attended school in this city [18] . The average school-going age is around 4–5 years but some schools also enroll very young children aged 1–3 years, usually belonging to families of high- or middle-income socioeconomic status. Therefore, this study included all children aged < 15 years and who have been injured in a school setting. The study duration was one year from July 1, 2007 to June 30, 2008.

### Measures

The surveillance study included all consecutive cases reporting to be injured and issued an ED slip for treatment for a nominal fee of 2 cents (in US$) in the selected hospitals. The World Health Organization’s (WHO) minimal data set questionnaire for injury surveillance [19] was administered after informed consent in a face-to-face interview to all the injury cases. The questionnaire included information on the following characteristics: age, sex, place (including schools), activity (playing, educational, others etc.), mechanism (falls, blunt object, sharp object, fire/heat etc.), nature (fracture, head injury, sprain, etc.), severity (mild, minor or superficial e.g. bruise or cut etc.; moderate, requiring some skilled treatment e.g. fractures or sutures etc.; severe, requiring intensive medical or surgical management e.g. internal hemorrhage, punctured organs, severe blood vessels etc.), and outcome of the injury case (discharged from the ED, hospital admission or death). This one-page questionnaire was translated into the local language Urdu and was back translated to English to ensure consistency.

### Data collection

Four data collectors from the hospital staff, supervised by a surgery resident, filled the questionnaires during 24 hours period. The questionnaires were then transferred to a central coordination office every third day. Two data entry operators entered the information on Excel spreadsheets. The principal investigators checked 10% of the data during coding and entry to detect errors during these steps. Informed consent was obtained from the parents or guardians of injury cases < 15 years. No information identifying individual cases was disseminated. Ethical approval of study methods were approved from the Institutional Review Board of the Rawalpindi Medical College and three teaching hospitals.

### Analysis

Only cases aged <15 years and who reported that an injury had taken place at school were selected for further analyses. Descriptive analyses of age, sex, mechanism, activity, nature and outcome of school-based injury cases were performed using Statistical Package for Social Sciences Version 15.0. Missing values were not considered while computing proportions. Chi-square tests were used where appropriate to assess characteristics associated with adverse injury outcomes i.e. hospital admission vs. the ED discharge.

## Results

### Sample

Out of 16,022 injury cases reported aged < 15 years, 923 (5.7%) occurred in schools. Response rates for different variables ranged from 93.0%-99.5%. The mean age was 8.3 years (standard deviation [SD] ± 3.3, range 1–14 yrs.). The most commonly affected age group was between 5–9 years of age (44.0%) (Table 1). Males suffered school-based injuries more frequently than females (73.8% vs. 24.8%) with the ratio being 2.9:1. There was only one reported fatality, a female child aged 8 years. This case was not considered in the following analyses.

**Table 1:** Description of children aged 0–14 years injured in schools (N = 922)*, Rawalpindi (Jul 2007 to Jun 2008)

	**All cases**		**ED discharge**		**Hospital admission**		***p***
	**N**	**%**	**N**	**%**	**N**	**%**	
Total	922†	100.0	861	95.0†	46	5.0†	
Age	*(n = 922)*		*(n = 861)*		*(n = 46)*		0.005
- 1-4	130	14.1	124	14.4	2	4.4	
- 5-9	406	44.0	385	44.7	14	30.4	
- 10-14	386	41.9	352	40.9	30	65.2	
Sex	*(n = 922)*		*(n = 851)*		*(n = 44)*		0.06
- Male	680	73.8	220	25.9	39	88.6	
- Female	229	24.8	631	74.1	5	11.4	
- No response	13	1.4					
Activity	*(n = 922)*		*(n = 854)*		*(n = 46)*		‡
- Playing	789	85.6	753	88.2	25	54.3	
- Educational/Academic	66	7.1	45	5.3	18	39.1	
- Other	60	6.5	45	5.3	3	6.6	
- No response	7	0.8	11	1.2	0	0.0	
Mechanism	*(n = 922)*		*(n = 836)*		*(n = 45)*		‡
- Fall	797	86.4	763	91.3	25	55.6	
- Blunt objects	26	2.8	26	3.1	0	0.0	
- Fire/heat	22	2.4	5	0.6	17	37.8	
- Sharp object	20	2.2	17	2.1	2	4.4	
- Moving vehicle	14	1.5	12	1.4	1	2.2	
- Suffocation	2	0.2	2	0.2	0	0.0	
- Drowning	1	0.1	1	0.1	0	0.0	
- Others	10	1.1	10	1.2	0	0.0	
- No response	30	3.3					‡
Type	*(n = 922)*		*(n = 859)*		*(n = 46)*		
- Unintentional	904	98.1	848	98.7	44	95.6	
- Violence	9	1.0	8	0.9	1	2.2	
- Self-harm	4	0.4	3	0.4	1	2.2	
- No response	5	0.5					
Nature	*(n = 922)*		*(n = 808)*		*(n = 43)*		‡
- Soft tissue injuries	284	30.8	279	34.5	3	7.0	
Limbs (muscular) §	273	96.1	269	96.4	2	66.7	
Facial & neck§	10	3.5	0	0.0	1	33.3	
Spinal§	1	0.4	10	3.6	0	0.0	
- Open wound	254	27.5	248	30.7	5	11.6	
- Bruise	117	12.7	116	14.4	0	0.0	
- Fracture	90	9.8	82	10.2	6	14.0	
- Head injury	64	6.9	52	6.4	12	27.9	
- Sprain	25	2.7	24	2.9	0	0.0	
- Burns	24	2.6	7	0.9	17	39.5	
- No response	64	7.0					
Severity	*(n = 922)*		*(n = 861)*		*(n = 46)*		<0.001
- Mild	648	70.3	629	73.1	7	15.2	
- Moderate	269	29.2	232	26.9	39	84.8	
- Severe║	5	0.5					

* The count is 922 as the only fatality was reported separately in text.

†% computed of total n = 907, missing observations n = 15.

‡ Chi square test not computed because of expected count <5.

§% computed for only soft tissue injury cases.

║ Regrouped with moderate injury.

ED – Emergency department.

### Injury circumstances

Around 85.6% (n = 789) of the school-based injuries took place during playing followed by during educational activity (n = 66, 7.1%). Falls (n = 797, 86.4%), striking blunt objects (n = 26, 2.8%) and fire/heat (n = 22, 2.4%) were the common mechanisms of school injuries. More than a third of school-based injuries during educational activities resulted from burns (n = 18; not tabulated).

### Intent of injuries

About 98.1% of these school-based injuries were unintentional in nature; however there were 4 (0.4%) students who harmed themselves deliberately while 9 (1.0%) injuries were intentional in nature and inflicted by another individual. Of these 9 injuries, 6 occurred as result of fight with peers. The object commonly used in these fights was stick (n = 5; not tabulated).

### Injury outcomes

Reported injuries frequently involved soft tissues (n = 284, 30.8%), or resulted in wounds (n = 254, 27.5%) and bruises (n = 117, 12.7%). Soft-tissue injuries were mostly muscular involving upper and lower limbs (n = 273, 96.1%). Around 70.3% (n = 648) of all the injuries were mild in severity while 29.2% (n = 269) suffered from injuries with moderate severity. There were only 5 (0.5%) cases where injuries were of severe nature. About 95.0% (n = 861) of the cases were directly discharged from the ED and about 5.0% (n = 46) required hospital admission; data was not available for 15 cases.

### Factors associated with adverse hospital admission

Some injury characteristics overrepresented in hospital admitted cases compared to ED discharge cases, which were; age 10–14 years (65.2% vs. 40.9%, *p* = 0.005), male (88.6% vs. 25.9%), injured during educational activities (39.1% vs. 5.3%), from fire/heat (37.8% vs. 0.6%), had burns (39.5% vs. 0.9%) and head injuries (27.9% vs. 6.4%) (Table 1).

## Discussion

This is the first study from Pakistan regarding the school injuries by using international surveillance methods [19]. Six percent of injuries in children <15 years reported in our ED sample occurred at school which is not insignificant. This proportion is less than one in five reported elsewhere but our sample included only the ED-reported injuries which itself is a significant outcome. This is consistent with another hospital-based study from Pakistan which showed that school injuries contribute towards 5% of unintentional injuries among children less than 11 years of age [20]. The National annual incident rate for school injuries in Pakistan is 1.6 per 1 000 per year [15]. Boys as the common gender, fall as the common mechanism and playing as the common activity were involved in school injuries which are comparable to what the literature reported earlier [21,22]. Most of the injuries were of minor severity however children aged 10 – 14 years were over-represented in hospital admissions compared to the ED discharges. This was also observed in other studies from both developed and developing countries [9,23,24]. Older children tend to engage in more risk-taking behavior leading to unintentional and intentional injuries [23].

Specific characteristics of the school environment may contribute to the likelihood of an injurious event. The panorama of the injury occurring in school has been described in low and middle-income countries (LMICs) [21,25]. Unfortunately, much less is known about the cause and the nature of injuries in developing countries. Ill-planned structure, inadequate recreation space and lack of proper playground equipment are among the major hazards that place children at risk for fall-related injuries in LMICs [26,27]. A significant proportion of schools in Pakistan is established in residential areas including apartments or houses. Based on literature and our experience we arguably suggest that inadequate playground equipment (e.g. rides) and inadequate supervision during sports may be the important factors involved in fall injuries; the most noted injury mechanism in our sample. Generally, there is no age standard for children to use rides; children tend to use rides which are not appropriate according to their height causing serious fall injuries leading to fractures of limbs. A study reported concrete and grass/soil surfaces [28] while other reported synthetic surfaces, for example wood and rubber were more involved in serious injuries [29]. A clear picture may emerge in future studies focusing on hazard assessments of playing facilities in these schools.

It is noteworthy that educational activities were associated with severe outcome i.e. hospital admission. We interpreted this category carefully as the WHO questionnaire did not distinguish between classroom and physical education activities. Cross tabulating activity with injury mechanism and nature suggested that one third of injuries during educational activities were burns occurring from fire/heat exposure; such might have occurred in the science or chemistry laboratory settings but could not be confirmed in this study. Utilization of adequate countermeasures and awareness of such mechanisms are perhaps low in Pakistan and therefore may need attention and investigation to develop more evidence-informed measures.

Although, the number of reported intentional injuries was very low in our study, it is important to understand the detail circumstances in which a child presents with such an injury because of its psychosocial impacts. The underreporting of intentional school injuries cannot be overlooked. Lack of supervision, younger age group and unstructured play might be some of the important underlying factors [23] but could not be reliably recommended based on our findings [7].

### Limitations

Despite a high response rate, the study has several limitations. First of all it included only cases presenting to teaching hospitals therefore it is likely that mild injuries were not reported in the hospitals. This study might have a selection bias towards severe forms of injuries that guardians or parents had considered their children to be treated at ED settings [30]. Secondly, the data might underestimate deaths as admitted patients were not followed till discharge. Thirdly, the description of injury was categorized on the basis of severity and multiple injuries were not taken into account. We intentionally however, avoided combining body part categories as this is a pilot study and making available details about injury might help in setting future research priorities. No a priori sample size estimations were possible as this was a descriptive analysis. Nevertheless, reported proportions might be useful in such estimations in future studies.

#### Conclusion and Recommendation

In conclusion, school-based injuries are not insignificant in Pakistan. Our pilot study suggested that detailed injury hazard assessments of schools and concerted advocacy efforts such as the legislation and enforcement of school safety standards in improving construction regulations within the schools are needed to effectively prevent such injuries. For this, a school injury surveillance system can be developed and linked to a designated hospital for that school. This may help in devising a mechanism for referral to a designated hospital for more severe injuries. [13]. Findings indicated the need to investigate staff-student ratio, availability of buildings, playgrounds, and basic first aid facilities, as well as safety-related lessons in school curriculum to inform school injury prevention policies in Pakistan [31].

## Consent

As recommended by the Institutional Review Boards of the Rawalpindi Medical College and the teaching hospitals, an informed consent was obtained from the parents/guardians of cases aged <18 years on prescribed forms about data collection and its use for research-related purpose. This report includes no images or personal information of any child.

## Competing interests

The authors declare that they have no competing interests.

## Authors’ contributions

URK helped in statistical analysis and writing of the draft. JB participated in the design of the study and performed the statistical analysis and helped to draft the manuscript. NZ helped in statistical analysis and writing of the draft. UF conceived the study, and participated in its design and coordination. All authors read and approved the final manuscript.
